# How older adults with mild cognitive impairment relate to technology as part of present and future everyday life: a qualitative study

**DOI:** 10.1186/s12877-016-0245-y

**Published:** 2016-03-31

**Authors:** Annicka Hedman, Eva Lindqvist, Louise Nygård

**Affiliations:** Department of Neurobiology, Care Sciences and Society (NVS), Division of Occupational Therapy, Karolinska Institutet, Fack 23 200, SE-141 83 Huddinge, Sweden

**Keywords:** Activities of daily living, Aging in place, Grounded theory, Mild cognitive impairment, Technology

## Abstract

**Background:**

Existing everyday technology as well as potential future technology may offer both challenges and possibilities in the everyday occupations of persons with cognitive decline. To meet their wishes and needs, the perspective of the persons themselves is an important starting point in intervention planning involving technology. The aim of this study was to explore how persons with mild cognitive impairment relate to technology as a part of and as potential support in everyday life – both present and future.

**Methods:**

Qualitative in-depth interviews with six participants aged 61–86 were conducted and analyzed, using a grounded theory approach.

**Results:**

The findings describe the participants’ different ways of relating to existing and potential future technology in everyday occupations as a continuum of downsizing, retaining, and updating. Multiple conditions in different combinations affected both their actions taken and assumptions made towards technology in this continuum. Both when downsizing doing and technology use to achieve simplicity in everyday life and when striving for or struggling with updating, trade-offs between desired and adverse outcomes were made, challenging take-off runs were endured, and negotiations of the price worth paying took place.

**Conclusions:**

Our findings suggest that persons with mild cognitive impairment may relate to technology in various ways to meet needs of downsized doing, but are reluctant to adopt video-based monitoring technology intended to support valued occupations. Feasibility testing of using already-incorporated everyday technologies such as smartphones and tablets as platforms for future technology support in everyday occupations is suggested.

## Background

Technologies are used increasingly in our everyday lives, offering new and more efficient ways to carry out many everyday activities [1]. However, the continuous development of new technologies also adds challenges, as efficient use often demands a skilled and habituated user. In persons with mild cognitive impairment (MCI) [2] these challenges are more evident than in older adults with no known cognitive decline, although not as pronounced as in persons with dementia [3]. For example, for persons with MCI, everyday technologies (ETs) at home and in public space, such as internet banking or ticket-vending machines or kiosks for travel, are generally observed and perceived as more difficult to use than for cognitively healthy older adults [3–5]. ET is however an inevitable part of daily life activities when still living at home, and is thereby necessary for older adults with MCI to relate to.

In light of the demographic changes with aging populations in many parts of the developed world, much hope is directed to future possibilities for aging well at home with support from ambient assisted living (AAL) technology [6]. The concept of AAL refers to integrated assisted living technologies based on ambient intelligence, i.e. digital environments that are sensitive, adaptive, and responsive to human needs. Applications potentially relevant for older adults with cognitive impairments include systems monitoring and providing reminders during activities of daily living (ADL) [7]. Despite being an evolving field, AAL technology also faces challenges regarding ethical considerations, computational issues, and low end-user acceptance. For example, studies examining end-user acceptance of video-based AAL systems have reported a gap between accepting the system in principle and actually being willing to use it themselves [8]. This points out the importance of exploring the needs and priorities of the end users early when developing assistive technology as well as ET [9, 10]. However, this has with few exceptions [11–13] rarely been the point of departure in technology development for persons with cognitive impairments. A systematic review of acceptance of technologies intended to support aging in place, for example safety monitoring and electronic memory aids, showed that beyond the characteristics of the older adults themselves, factors influencing acceptance could be divided into concerns regarding expected benefits of, perceived need for, alternatives to, and social influence of technology [14]. The reviewed research did not cover ET, and seldom included technology supporting instrumental activities of daily living (IADL), or the voices of people living with cognitive decline. These aspects are encompassed in this study.

As part of an international project to investigate the potential of AAL technology for older adults with MCI [15], this study explored how persons with experience of living with MCI relate to both technology presently incorporated in their everyday activities, and potential future assistive technology. As the starting point was the current activities and roles identified by the participants themselves as important, this broad approach regarding technology in everyday life was chosen. Furthermore, the boundaries between ET and assistive technology are becoming increasingly blurred. ETs such as smartphones and tablets are known to be customized by users themselves and their significant others, and technological systems and products are combined in efforts to fit personal needs, and regarded as potential platforms for assistive and AAL technologies [16–18]. Therefore it is relevant to jointly address the potential of different types of technologies to support present and future everyday activities in older adults with MCI. This study aimed to explore how persons with current or recent experience of living with MCI relate to technology as a part of present and future everyday life.

## Methods

### Design and setting

In this qualitative study six persons living in an urban area in Sweden were interviewed twice each in their homes. As the design was inspired by grounded theory [19], inclusion of participants, data collection, and analysis took place simultaneously. This meant that emerging findings and gaps detected in the data continuously guided data collection.

### Participants

Sampling took place from an initial sample of 37 persons with MCI that had been followed for four to five years in a longitudinal study [20–22]. This provided a unique opportunity to supplement longitudinal questionnaire-based information about perceived ET use and activity involvement with detailed qualitative data on how persons with MCI relate to technology in their everyday activities, both present and future. Our sampling frame of persons with stable MCI was limited, mainly due to diagnostic changes within the initial sample, which reflects the natural course of MCI. Therefore we decided that persons who had recently either progressed to dementia or reverted to normal cognition, were also eligible for inclusion. Accordingly, the inclusion criteria for the present study were: (a) fulfilling, or having fulfilled within the past two years, the Petersen MCI criteria [23], i.e. subjectively perceived cognitive decline which is objectively verified in clinical assessment, no dementia, and overall intact ADL/IADL; (b) being ≥55 years; (c) being a user of ET (that is, not being totally dependent on assistance from others in daily life activities), and (d) being able to participate in interviews. Within the sample followed prospectively 13 persons fulfilled inclusion criteria (a), (b) and (c), but six of these 13 persons did not fulfil criterion (d). After inclusion of and interviews with six participants, data was considered sufficiently rich to reach the aim of the study. Demographic characteristics of the participants at inclusion, and longitudinal information regarding changes in clinical and functional characteristics are presented in Table 1.

**Table 1:** Demographic data and information on current diagnostic and cognitive state, activity involvement, and everyday technology use, including retrospective changes

Aliases in order of sampling	Gender	Age	Marital status	Current vocational activity	Profession	Diagnosis (time with current)	MMSE^a^, (change score^d^)	FAI^b^, (change score^d^)	ETUQ items used^c^, *n* (change score^d^)	GDS^g^ (score)
Albert	Male	86	Widower	Retired	Farmer	AD^e^ (6 months)	24 (−2)	22 (+2)	26 (±0)	2
Brita	Female	78	Married	Retired	Economist	MCI (5 years)	26^f^ (−4)	22 (−13)	28 (–34)	3
Caesar	Male	78	Married	Retired	Engineer	MCI (4 years)	28 (−2)	30 (−2)	56 (−12)	2
David	Male	74	Married	Retired	Engineer	MCI (5 ½ years)	28 (±0)	26 (−2)	59 (+5)	2
Eric	Male	61	Married	Full-time sickness benefits	Social worker	MCI (5 ½ years)	29 (−1)	27 (−10)	64 (+10)	8
Frida	Female	63	Single	Half-time working, half-time sickness benefits	All-around job in a catering business	No known cognitive impairment (3 ½ years)	29 (±0)	35 (+2)	56 (+1)	4

Notes:

^a^Mini-Mental State Examination has possible scores between 0–30. Higher score indicates better cognitive status [39]

^b^Frenchay Activities Index has a scale ranging from 0–45. Higher score indicates more active lifestyle [40]

^c^Indicates how many of the 92 items in the Everyday Technology Use Questionnaire [3] have been used in the past 12 months

^d^Change scores refer to differences from baseline in retrospective 5-year data, except for Albert and Caesar where 4-year data is provided

^e^Alzheimer’s disease

^f^Could not complete the 1-point drawing task in MMSE due to severe hand tremors

^g^Geriatric Depression Scale has a possible total score between 0–20. A score exceeding five indicates possible depression [41]

### Data collection procedures

The interviews, each 30–90 min long, took place between June 2013 and April 2014. They were divided into two sessions for each participant to allow rich in-depth data, yet avoid lengthy and tiresome interviews. In total, 11 h and 38 min of interview data were audio-recorded, and field notes were taken. During the interviews an interview guide was used. It was first pilot-tested with an older adult without cognitive impairment to ensure that the question areas prompted reflections relevant to the study’s aim. The guide focused the first interview on activities, interests, habits, and roles that were important in the participants’ daily lives, why these were important, and on changes – actual and/or anticipated – in how the participants managed to carry out these activities and habits and fulfill these roles. The second interview focused on how the participants related to current and potential future technology, and on views of themselves as technology users in daily life. The point of departure in the second interview was important activities they had discussed in the previous interview, and questions addressed, for example under which circumstances they would accept monitoring, if needed to support valued activities.

### Data analysis

The analysis was guided by the grounded theory approach elaborated by Charmaz [19]. Directly after the initial interviews each audio recording was listened through by the first author. Verbatim transcription of interviews by research assistants, initial coding, and inclusion of new participants thereafter took place simultaneously. Initial coding was performed line-by-line or sequence-by-sequence by the first author with the intention of constructing codes that were close to the data, capturing actions. Use of the NVivo 10 software for Windows facilitated structuring these codes, supporting constant comparisons within the data, which spurred merging or splitting of codes. In parallel longer memos were written to capture early analytic ideas. When the initial coding was completed for all data, questions that highlighted the aim of the study from different perspectives were posed to the data to help focus the analysis on how the participants related to technology in their present and future activities. Questions included: (a) How do the participants view the possibility to maintain important activities, habits, and roles by support from technology? (b) Under what conditions do the participants consider using technology as support, or not? (c) What data is at hand regarding maintaining or refraining from activities, habits and roles where technology comes into play as a complication or support? Data answering these questions were extracted, sorted, and discussed among the authors. To create an overview and further focus on the data regarding technologies, mind-mapping was also used [19], where each participant’s views on and ways to relate to technology were outlined. These steps helped to identify significant leads in the analysis. By continued comparisons of the properties within these leads, three distinct yet related categories, which characterized the participants’ ways of relating to technology, were identified.

## Results

The analysis revealed three different ways of relating to technology – *downsizing*, *retaining,* and *updating* – presented in more detail below. These ways were influenced by promoting or impeding conditions as well as trade-offs between desired and adverse anticipated outcomes. Multiple *conditions* in various individual combinations affected whether the participants changed or retained use of existing technology, and whether they considered or rejected use of potential technology. These conditions included the participants’ perceived need for change, whether they currently struggled with or envisioned their own future decline, the importance and habituation of the activities in question, whether they assumed that technology could support valued activities, and finally the availability of alternative solutions. *Trade-offs* raised by the participants concerned integrity, safety, facilitating vs. training, impact of the technology on their spouses, and costs. Additionally, *take-off runs* often had to be endured before the hoped-for usefulness of the technologies was realized; successfully passing these was often dependent on support from friends and family. The paradox of having to pass difficulty to achieve simplicity was recurrent and negotiations of the price worth paying were ever-present.

### Relating to technology in everyday life by downsizing, retaining, and updating

**Fig. 1:**
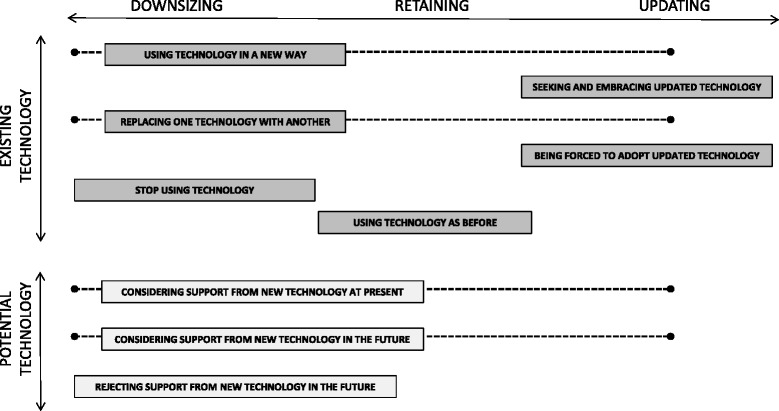
Overview of the categories and subcategories describing ways of relating to technology in everyday life

The analysis showed that the participants’ different ways of relating to existing and potential future technology in everyday life could be described as a continuum from downsizing, through retaining, to updating. For each part of this continuum distinct ways of relating to technology appeared in actions taken towards *existing technology* or assumptions made about *potential future technology* use (see Fig. 1).

Most participants described ways of relating to technology in all three parts of the continuum. That is, in parallel to downsized doing and technology use, most participants also exhibited retained doings and ways to relate to technology, as well as doings and ways of relating to technology suggesting either a quest or need for updating. Furthermore, some ways of relating to technology contained qualities of downsizing, retaining as well as updating; these floated along the continuum as indicated by the dotted lines in Fig. 1. The ways of relating to technology are presented in the following in connection with what was interpreted as the main quality, based on the intention of the actions taken or assumptions made by the participants.

### Downsizing

Changing abilities had forced the participants to develop different downsizing approaches in their everyday activities, for example simplifying by accepting help from others and sticking to familiar activities. The need for downsized doing also showed in and affected the ways the participants related to technology. Downsizing (Fig. 1) thus delineated both their ways of relating to doing in general and ways of relating to existing and potential future technology in everyday activities.

#### Using technology in a new way

Two different kinds of actions taken were identified when using existing technology in a new way due to a need of downsized doing. Firstly, some participants *started using services possible to activate in technologies already in use* to simplify doing or to increase safety. For example, Caesar, David, and Eric were responsible for paying the bills in their households, which in Caesar’s case also included his older sisters’ finances. They had long been using internet banking, but more recently made efforts to downsize the activity by shifting to direct debit and electronic invoices to make the payment of bills less complicated. As this shift included incorporating new simplifying services, it added an updating quality to using technology in a new way. Implementing the steps required before a new simplifying service could be used in existing technology was an example of a take-off run, often challenging and presupposing a savvy user. The goal – the hoped-for usefulness – needed to be sufficiently motivating for these take-off runs to be endured, suggesting that there was a price to pay.

But of course, if you have reduced capabilities and concentration, and so on, then this motivation bit has to be stronger if you’re going to make it all work. That’s the way it is. That you kind of feel that you’ve got to do it, you know. Otherwise, well … it’s not going to work otherwise. (Eric, 61 years)

A second identified way of using existing technology in a new way was to *use it more seldom*. In Brita’s case this came about naturally due to changed habits; she seldom used her mobile phone and travel card nowadays as she most often stayed at home. It could also be a conscious strategy, as for Eric who had stopped watching TV with his wife in the evenings. Knowing that he at that time of the day did not have enough energy left to follow the plot of a TV program, he instead chose to go to bed early to read something “easily digestible”.

#### Replacing one technology with another

When the participants experienced difficulties with existing technology they sometimes acted by replacing use of one technology with another. Albert explained that as he could no longer drive a car, he had switched to using a bicycle or commuter train. Instead of using e-mail as before to keep in contact with a relative living abroad, Brita now used the landline phone and the relative in turn wrote letters. Brita’s memory-related difficulties and hand tremors were only two of several reasons prompting this change. Caesar had replaced his former mobile phone with a new simplified senior mobile phone. He really wanted to recommend other elderly to do this; with the simplified senior mobile phone Caesar had learnt how to send text messages, which he never had managed with his former mobile phone. This exemplifies how downsizing by reducing difficulty in doing through shifting to a simplified product also entailed updating elements.

#### Stop using technology

In a few cases the action taken was to stop using an existing technology when declining abilities led to either difficulty in using the technology itself or difficulty in performing the specific activity. When her cognitive impairments debuted Frida had tried using the Google calendar for reminders. However, she experienced the reminders as very stressful and switched back to using her usual pocket calendar, despite having recurring problems remembering to check it, and thereby occasionally missing scheduled commitments. Another example was that Brita had stopped using internet banking when her son assumed responsibility for handling the bills. She explained:

He probably thought that it was difficult for me. […] But I thought, I guess I shrugged my shoulders a little and thought “If he wants to do this, I think that’s fine, then I don’t have to do it”. No, but I’ve got so much else I want to do, so it’s great! (Brita, 78 years)

Brita seemed to experience it as a relief and even a privilege to have another person available who could free her from this task. She reasoned in a similar manner regarding her daughter-in-law taking over much of the housework, such as cooking and cleaning, which made Brita stop using household technologies like the vacuum cleaner. She noted that these arrangements made it possible for her to do whatever else she wished, although regretfully she added that many of her plans were not realized.

#### Considering support from new technology at present

When relating to potential technology a few participants considered adopting new technology for support even at present to be able to simplify and downsize their doing and thereby facilitate performance. This downsizing way of relating to doing thus also entailed updating technology. Conditions for taking this stance were that they currently struggled with problems in doing that they envisioned could be solved by technology support. However, trade-offs were made regarding the risk of losing abilities if simplifying doing with technology, and also regarding integrity issues. For example, Brita thought that a technical solution that could offer her different proposals to choose from would currently be a valuable help in decision-making, and Frida thought that step-by-step instructions from an integrated technology might be useful even at present, for example when baking. But if such prompting solutions implied camera monitoring, both were presently reluctant. Allowing camera monitoring in one’s home was a price no participant was prepared to pay at present, and the reasons were that no urgent need of instructions was perceived, that integrity would be threatened, and that one would become overly aware of one’s own mistakes. A trade-off regarding integrity raised contradictory requirements on a potential voice recorder for memory support. Brita wanted a memory support that would “call out to her sometimes”, if not, she suspected that she would not get around to using it. At the same time Brita stressed that it was important that the memory support was discreet; she would otherwise feel embarrassed in front of others and would restrict its use to her home.

#### Considering support from new technology in the future

Commonly the participants related to potential technology by considering adopting it for support and compensation in the future; this again also entailed updating. Envisioning possible future scenarios with progressively reduced ability and increasing need to downsize doing was especially evident in Eric’s case. Eric stressed that although it was important for him to do things himself he regarded future downsized doing supported by technology and/or other persons as a better option than not being able to do activities at all. He showed great faith in the potential of customized technology solutions, and speculated on how his great interest in downloading music via the Spotify music-streaming service could be possible to sustain in the future:

If you take me as an example here, that I think *music* is fun. And like then you have to *adapt* these things, I’ll never manage *this*, with Spotify and all that. If…if I get a little more scatterbrained, eh? That won’t work. There has to be some basic-Spotify variation of it all, right? And you have to solve that, then. So that I, then I can get access to my Spotify list just by pushing two buttons. (Eric, 61 years)

He also realized that in addition to a future need of simplifying the streaming application presently used, the activity itself would likely have to be downsized; this was a price that he currently considered would be worth paying.

However, despite being beneficial in some ways, simplifying technology was also regarded by some participants as potentially risky for the preservation of one’s abilities. Use of prompting technologies during activity performance was assumed to entail the risk of becoming a passive recipient dependent on technology, and forgetting things once known.

The disadvantage is maybe that you don’t exert yourself enough to figure out how to do it. (Albert, 86 years)

The ways of relating to potential technology involving monitoring or GPS tracking were also characterized by trade-offs about integrity; typically the views of the participants depended on what was at stake. Some, but not all, assumed that they would revise present cautious views and accept monitoring technologies in the future should their abilities deteriorate to the extent that this became necessary for their own safety. Here they referred to fall detection, for example, and possibilities to move freely outdoors despite problems finding their way, or if activities such as cooking otherwise became impossible to perform.

Yes… No, that [integrated prompting technology with computer vision] is something I’d like to avoid. Anyway, in the shape I’m in *now* I’d like to avoid it. But I can imagine that sometime one might *need* it… and then maybe one would appreciate it. And think it’s good. (Albert, 86 years)

On the positive side of the integrity trade-offs Albert mentioned that support from technology can be useful if you want to hide your difficulties from other people. However, those who would consider future monitoring were concerned about its impact on their spouses. Brita, for instance, knew her husband had a strong sense of integrity, and Eric did not want his wife to feel obliged to check on him while she was at her workplace.

#### Rejecting support from new technology in the future

For different individual reasons some participants rejected potential future support from prompting technology in their homes if monitoring was a prerequisite for it. The way of relating to prompting technology was characterized in Caesar’s case by envisioning alternative solutions and underplaying the problems experienced, while David was doubtful that prompting would support the activities he valued. Trade-offs around integrity were common. Envisioning alternative solutions and underplaying difficulties were exemplified by Caesar, who despite reporting some cooking problems expressed no willingness to incorporate monitoring technologies for prompting and safety to facilitate this activity in the event of future worsened cognitive state. He suggested alternative simplifying solutions, such as giving up his disorganized recipe collection and instead starting Googling recipes, or buying timers for the coffee machine and stove instead of monitoring for safety. By stressing that such solutions were also beneficial for persons without cognitive impairments and that they may experience the same needs for such simplifications, Caesar seemed to underplay his own potential future decline when prompting might be helpful. David dreaded the possibility of further cognitive decline, and doubted that prompting technology in the future could support the activities he valued most, including the intellectual exchange with friends. In their trade-offs of being monitored, integrity outweighed safety and made some participants reject this technology for the future.

There were also examples of activities that were abandoned, or that one was afraid of losing, where supporting technology solutions were difficult to imagine. There was no obvious possible price to pay involving simplification through technology that would enable a downsized way of doing these activities. This situation involved activities relying on and requiring performance capacities that had now declined, or were perceived as at risk of declining. These activities were typically performed without technology, such as playing musical instruments, reading books or newspapers, taking outings, or having intellectual exchanges with friends. Especially Albert, being the oldest participant, seemed to reconcile himself to these non-negotiable downsizings, embracing them as a natural part of aging. Others, like Brita and Frida, expressed a longing for what had been lost, and David, despite not yet having faced such forced downsizing of doing, stood out as very worried about the future, envisioning a coming catastrophe.

And … (pause) the next step is probably that it can be difficult to pay a cashier, what do I know? But it’ll become successively so that what’s simple and natural now, that you can find to be foolishly done or like that, it becomes *impossible*. Then you just can’t do it! […] Yes, but *everything* like that will be destroyed. It’ll be, it’s a *catastrophe*! (David, 74 years)

### Retaining

In between downsizing and updating, an intermediate, sometimes fragile, situation of retaining technology use and doing in everyday life could also be found. Retaining (Fig. 1) often concerned technologies mentioned in passing when participants described their everyday activities, that is objects that were self-evident parts of everyday life, and used without reflection. However, these technologies could despite their mundanity be prerequisites for central habits. Furthermore, some participants consciously maintained use of specific technologies to retain doing that worked well enough, suggesting a need to preserve a delicate equilibrium to maintain seamless everyday doing. This more conscious retaining approach often implied a reluctance to adopt updated technology, thereby avoiding challenging take-off runs.

#### Using technology as before

The participants chose to *use technology as before* when it was well incorporated in their habits, when it met their needs, when the cost of adopting new technology – financially or in effort – was perceived to be too high, and when no added value was expected by changing to updated technology.

An example of a central habit supported by continued use of an everyday technology was David’s morning routine of listening to the radio before taking a walk with a neighbor. During these walks they discussed the topics just debated on the radio. This exchange of ideas was central to David and exemplified what he was most worried about losing should he develop dementia. Another example was Eric’s well-established habit of barbecuing familiar dishes on the gas grill during the summer.

Technology also continued to be used as before if it fulfilled its purpose and met the participants’ needs. For example, for David and Frida the limited functions of their mobile phones, which could be used only for calls, were sufficient. Avoiding the updating of current technologies that supported one’s everyday activities sufficiently well to avoid the take-off run inherent in new learning was a common approach mentioned by the participants. For example, Eric believed that twenty years ago he would probably have considered keeping up with new trends around barbecuing, but now he thought it just seemed like a hassle and preferred to stick to the familiar gas grill. Frida’s lines of thought were similar concerning her decision not to replace her somewhat slow computer or her digital camera now that she had learnt their functions.

Continued use of current technologies was also preferred if no benefits were identified in updated alternatives. David had never tried to use a self-service checkout station in a store, as he saw no added value in it. Rather, he anticipated it to entail extra work for him in addition to probable malfunction of the technology.

### Updating

Finally, the analysis revealed that some participants were also open to challenging themselves and expanding their activity repertoires, despite the difficulties they experienced. This quest for updating and renewal occurred in parallel and in contrast to seeking out and appreciating well-known activities and routines, and was visible in activities performed both with and without technology. Updating (Fig. 1) comprised both a strong quest and a forced need for renewal of doing in general and typical ways of relating to updated technology; some participants sought such technologies, while others on the contrary struggled with the technology pressure.

#### Seeking and embracing updated technology

The analysis showed that the participants related to updated technology, i.e. new technology without the primary purpose of compensating for declining abilities, in different ways under different circumstances. Conditions met when the participants *sought and embraced updated technology* were that the technologies fitted and supported important activities and roles, entailed added values, and were perceived as affordable. Additionally, availability of help in the social network was often crucial for being able to install or learn the new technology. An example where these conditions were met was the new tablet that Caesar had received as a gift from his children. He used it daily for playing an online crossword game, involving all his children and a daughter-in-law. Playing the game on the tablet was described as being fun, connecting him with his children on a daily basis, and potentially training his memory. This determination to train the brain was also seen in some participants in other ways of relating to technology, often leading to a hesitation to consider supporting and simplifying present and future technologies.

Seeking and embracing updated technology that fitted valued interests could open doors to new worlds. Eric described how he by subscribing to a play-list on a music-streaming service came to broaden his music interest to encompass new genres. Following the play-list had evolved into a hobby, triggering Eric to update his existing technology use by appropriating, learning, and incorporating several new technologies and services. These included downloading the music service app, learning to subscribe to the specific play-list, saving the music he liked as play-lists of his own, buying a docking station to listen to music via smartphone or tablet in the kitchen while cooking, and learning from his son how to run the docking station wirelessly.

But what I can *do* then, is to listen to new music. So that I don’t *just* go on plodding through my old seventies music, like I’ve done… I listen a lot to ballad music, and some classical, and like that. And I’ve done that for many years, without updating myself. Now I think it’s sort of fun. But I’ve never listened to Arabic or African music, I think it’s very cool to *find out* that I like it. […] There’s like a combination of new technology that hasn’t been all that impossible to learn, and an interest in *broadening* a so-called *normal* music interest to like listen to a few other things, right? (Eric, 61 years)

However, the participants also stated that incorporation of new technologies required time and effort; the take-off run could be long and strenuous. A stance they shared was not being interested in the updated technology itself, but in the opportunities it offered. In order to reach the advantages offered by technology the participants realized that the take-off run needed to be endured, and managed.

#### Being forced to adopt updated technology

In addition to situations where the participants actively sought new technology, there were situations where they experienced that specific technologies imposed themselves on the participants, being impossible to avoid. Conditions that forced them into adoption of updated technology or new services in existing technology included when this was required to make a valued or needed activity possible, when other persons depended on one’s use and knowledge of the technology, when the updating was influenced by events beyond one’s control, or when technology was acquired as a gift. Challenging take-off runs in these situations included getting installations of new technology right, implementing steps required before a new service could be used in existing technologies, or learning to use the technology. The period preceding the actual mastery of the technology they were forced to adopt was described as “damned tough”, and was at times something they worried about.

Examples of forced updates beyond Frida’s control that were required to perform her work tasks and that her colleague relied on her to master, were a new booking and payment system on computer and tablet, a new alarm system, and a new smartphone soon to be introduced at her workplace. Frida worried about these upcoming novelties, and the situation even made her son suggest that she should give up her part-time job and retire. She lacked the opportunity to opt out of learning this range of updated technologies, but was unsure whether the work situation would tolerate her slow way of “cramming in” new knowledge.

Sometimes this forced technology updating challenged the participants by introducing difficulties when doing the activities they wanted and needed to do; to resolve the situation and shorten the take-off run they often had to summon help from friends, children, or grandchildren. For example, a dilemma for Eric concerned the mandatory updates of software such as Skype or image-processing software he used on the computer.

And I know, photos that I’m interested in, right? Then it’s always like when you’ve taken them and are going to empty the memory card and put the pictures in the computer, then they’ve *always* upgraded my image processing system, which is a very basic program that belongs to the camera, because I don’t work with pictures so much, but I just sort them, and download them to a disc then, so I’ve copied it. But… then they’ve made changes in this program that I’ve like… And it feels rather as though someone has moved the furniture around *at home*! That like, that I don’t want. And if I reject this, then I can’t process the pictures. You just can’t manage it, instead you have to sit and re-learn […] I mean, it’s the same thing as if they were to upgrade my car every time I use it. That new buttons and gauges were added as soon as you wanted to go out and drive it. You’d be bothered by that, like, “My gosh, doesn’t it work as it should?”. (Eric, 61 years)

These updates destroyed the familiar interface and suddenly made his habitual mode of use non-applicable. Oftentimes the updates were impossible to ignore; they were the price he had to pay to be able to continue the activity he wanted to do.

## Discussion

This study identified a variety of ongoing parallel ways in which the participants related to existing and potential technology as support in their everyday activities. Their ways of relating were characterized by present and assumed future actions to downsize, retain, and update everyday doing and technology use (Fig. 1). Interestingly, most participants reported ways of relating to technology in all three parts of this continuum, and furthermore their reflections and actions often involved combinations of downsizing, retaining, or updating qualities, as the dotted lines in Fig. 1 indicate. The findings underscore that technology is a complex and many-faceted part of everyday life that has great impact on the activities of older adults with cognitive impairments. For example, our findings show that persons with cognitive impairment may have a need and/or desire to update their technology use, while simultaneously striving towards downsizing. This nuances our earlier findings regarding use of less ET objects over time in the sample with MCI [21] of which these participants are a part. It also challenges the commonly held stereotype image of older people with cognitive impairments as being uninterested in updated technology, and has implications for health-care practitioners (such as occupational therapists) and providers of assisted living technologies.

In the interviews few participants told of examples when they had downsized doing by ceasing to use technology. Retrospective questionnaire data (Table 1) on the number of ET items that they used generally confirmed this, but also revealed a considerable decrease during the past five years in Brita’s case. Caesar, who had moved to smaller quarters, also used less ET objects. However, the participants’ numbers of ETs used over the past years were more often stable or slightly increasing, which echoes the present qualitative findings of also relating to technology by retaining or updating. Interestingly, the need and/or desire found in several participants to adopt updated technology contrasts with previous findings in environmental gerontology research of old people as being mainly reluctant to acquire new technical objects and systems, but rather considering to sort out objects from their material rooms [24]. This contrast between findings is particularly interesting as the participants in the study by Larsson Ranada & Hagberg, albeit older than the participants in our study, were cognitively healthy. Another notable difference is that we use the concept of downsizing in a wider sense than other authors [24, 25]. By downsizing we denote not only cessation of using technologies, but also a downsizing of doing which may even include incorporation of new technology for support. This wider use of the term was grounded in the participants’ data of a more scaled-back way of doing activities involving technologies, and served as a way of contextualizing technology in the doing.

The identified conditions and trade-offs that influenced the participants’ different ways of relating to technology are in line with the themes found in a review on acceptance of aging-in-place technologies in community-dwelling older adults. These themes included individual characteristics of the older adults themselves, their concerns regarding, expected benefits of, perceived need for, alternatives to, and the social influence of technology [14]. However, with our wider scope of also including existing ET, the present study adds new knowledge beyond acceptance of potential assistive technology to also address conditions and trade-offs for maintaining or changing use of already-adopted everyday technologies. This is interesting, as the presence of technologies in older adults’ homes is steadily increasing. For example, in 2014, 65, 43, and 27 % respectively, of Swedish persons aged 65–74 years, used a laptop, a smartphone, or a tablet to access the internet [26]. In our study all participants but one accessed and used a computer, and some were also frequent users of smartphones and tablets. Several participants showed readiness to use these technologies in new ways to meet their experienced needs of downsizing doing. However, this meant facing initial take-off runs where the availability of support from significant others oftentimes was decisive.

One condition identified was the importance of experiencing a need for change in a valued activity for the participants to alter the way they related to technology, both regarding downsizing and updating. When use of existing technologies worked sufficiently well and met current needs, present use was retained. Importantly, the need for change was also weighed against the price they had to pay in complex trade-offs. Similarly, an intervention study involving persons with mild Alzheimer’s disease found that the experience of an unmet need in a highly valued activity was a necessary starting point in the process of becoming a user of assistive technology for cognitive support [27]. A decision-making process at several junctures followed, involving for example decisions to adjust everyday routines, and to trust the assistive device [27]. The conditions and trade-offs identified in the present study addressed similar negotiations and decision-making. It is likely that these conditions and trade-offs are not unique to persons with MCI. Rather, they can be assumed to reflect a general human way of relating to technology in everyday doing. What may be more pronounced in persons with MCI is a greater vulnerability in the take-off runs encountered in the interactions with technology [28].

However, intervention studies indicate that persons with MCI may have untapped resources – possibly modulated by cognitive reserve factors – and ability to learn new strategies that facilitate daily life [29]. Another study suggests that persons with MCI favor momentary strategies, manifested as an intertwined process of learning and doing [28]. Interestingly, the participants in our study often spontaneously found, or assumed to find, ways to meet their needs of change arising with declining abilities by ways of relating to technology. These ways included using technology in a new way, replacing one technology with another, considering support from new technology at present or in the future, or using technology as before, as illustrated in Fig. 1. But situations when this was not the case were also exemplified; technology was not regarded as a panacea [17] and the participants told also of activities where technology support was difficult to imagine, or even undesired. Furthermore, the participants in this study tended to consider support from prompting technology only further ahead in time, while presently being reluctant. Their concerns and complex trade-offs regarding integrity issues and potential loss of capacities if simplifying their doing with technology have implications for designers of AAL technology. For example, a prompting AAL technology needs to provide just the right type and amount of support at just the right moment, in order to avoid interfering with the doing that still works. A dilemma, however, is that such balanced support would require advanced monitoring of daily life activities by intelligent assistive devices [30], to which the participants in this and earlier studies oftentimes are resistant [14, 31]. The ambivalence of feeling good *and* bad about potential future monitoring sensors – rather than good *or* bad – in relation to independence versus security and privacy versus intrusion has been noted [31], and was in this study exhibited in several participants. Peek and colleagues [14] also note that reluctance towards adopting such technologies for aging in place may be understood as strategies of the older adult for coping with decline. Such coping strategies may include conscious decisions not to focus on one’s future vulnerability in order to retain self-respect [32], but on the contrary to focus on the present [33].

An important contribution of this study is making visible under which conditions older adults with cognitive impairment may seek and embrace updated technologies and find great pleasure in using these in new engaging activities. The importance of a supportive environment in the first explorative phase of any change process has been theoretically emphasized [34], and was in this study empirically demonstrated in the premise of available support from other persons. Furthermore, the fit and support of a new technology to valued interests and roles to be worth the efforts of the take-off run, illustrate the profound motivating drive of enjoyment and sense of obligation [34, 35].

### Methodological considerations

Our choice to investigate how the participants related to both well-known ETs present in their lives and potential future AAL technologies has both strengths and weaknesses. An advantage is that this made it possible to focus on the activities the participants reported as central in their lives, and link both present and potential future ways of relating to technology for these activities. A disadvantage of mixing views of these different types of technologies in the same study is that they might hold different symbolic meanings for the participants [36] and be acquired in different ways. A narrower scope might have allowed further depth and focus of the findings, yet the overlaps between ET and assistive technologies are nowadays large [17, 18] and it is uncertain whether the participants would distinguish between these frameworks as professionals do. The number of participants in this study was restricted by the number of persons found eligible in our initial sample; this limited the potential to capture a rich variation in ways of relating to technology. However, variation was sought regarding age, gender, vocational background, and interest in technology, which was facilitated by our previous knowledge of the eligible persons.

## Conclusion

Our findings suggest that persons with MCI may develop their own creative solutions to meet needs of downsized doing, which simultaneously may involve downsizing, retaining, and updating technology use. However, updating oftentimes implied challenging take-off runs for the participants, which calls for health care practitioners’ attention, especially when support from significant others is lacking. The reluctance of many participants to adopt video-based monitoring technology, suggests that a preferable way forward may be to investigate the feasibility of using already incorporated ETs such as smartphones and tablets as platforms for AAL technology intended to support continued valued doing.

### Ethics approval and consent to participate

In this study we followed the approaches that have previously proven successful to enable informed consent from persons with mild dementia or MCI. These recommendations include using simple language, reducing information load, repeating information [37], and viewing the process of obtaining consent as continuing over the whole research period [38]. We also stressed that participation was voluntary and possible to discontinue at any time. Potential participants’ ability to provide informed consent was discussed and assessed jointly in the research team, based on our previous knowledge of the eligible persons from recent data collection in a longitudinal project. All persons who were invited agreed to participate. The study as well as the described consent procedures were approved by the Regional Ethical Committee in Stockholm (Dnr 2013/617-32).

### Consent for publication

Not applicable.

## Abbreviations

AAL: ambient assisted living
AD: Alzheimer’s disease
ADL: activities of daily living
ET: everyday technology
ETUQ: Everyday technology use questionnaire
FAI: Frenchay activities index
GDS: Geriatric depression scale
IADL: instrumental activities of daily living
MCI: mild cognitive impairment
MMSE: Mini-mental state examination
